# Dietary Impact of Adding Potassium Chloride to Foods as a Sodium Reduction Technique

**DOI:** 10.3390/nu8040235

**Published:** 2016-04-21

**Authors:** Leo van Buren, Mariska Dötsch-Klerk, Gila Seewi, Rachel S. Newson

**Affiliations:** 1 Unilever R&D Vlaardingen, Olivier van Noortlaan 120, Vlaardingen 3133 AT, The Netherlands; Mariska.Dotsch@unilever.com (M.D.-K.); Rachel.Newson@unilever.com (R.S.N.); 2 Unilever Germany, Knorrstrasse 1, Heilbronn D-74074, Germany; Gila.Seewi@unilever.com

**Keywords:** salt, sodium, sodium reduction, potassium, reformulation

## Abstract

Potassium chloride is a leading reformulation technology for reducing sodium in food products. As, globally, sodium intake exceeds guidelines, this technology is beneficial; however, its potential impact on potassium intake is unknown. Therefore, a modeling study was conducted using Dutch National Food Survey data to examine the dietary impact of reformulation (*n* = 2106). Product-specific sodium criteria, to enable a maximum daily sodium chloride intake of 5 grams/day, were applied to all foods consumed in the survey. The impact of replacing 20%, 50% and 100% of sodium chloride from each product with potassium chloride was modeled. At baseline median, potassium intake was 3334 mg/day. An increase in the median intake of potassium of 453 mg/day was seen when a 20% replacement was applied, 674 mg/day with a 50% replacement scenario and 733 mg/day with a 100% replacement scenario. Reformulation had the largest impact on: bread, processed fruit and vegetables, snacks and processed meat. Replacement of sodium chloride by potassium chloride, particularly in key contributing product groups, would result in better compliance to potassium intake guidelines (3510 mg/day). Moreover, it could be considered safe for the general adult population, as intake remains compliant with EFSA guidelines. Based on current modeling potassium chloride presents as a valuable, safe replacer for sodium chloride in food products.

## 1. Introduction

High dietary sodium intake is associated with an increased risk of hypertension, which is a risk factor for the development of cardiovascular disease [1,2]. The World Health Organization (WHO) recommends that adults consume less than 2000 mg of sodium daily (5 grams of salt) [3]; however, average global intake exceeds this level by far. Impact modeling shows that realistic reformulation of food products to lower sodium levels could decrease sodium intake by up to 30% [4]. Population-based studies have also demonstrated that dietary sodium reduction towards the WHO guidelines could lead to a significant reduction in blood pressure and cardiovascular disease risk [4,5,6,7,8,9,10]. Therefore, reformulation of food products, and reduction of discretionary salt usage, would contribute to a disease risk reduction [1,2]. In light of this, international health authorities advocate sodium reduction as a cost-effective strategy to improve public health [11].

The food industry is actively engaging in reducing the sodium content of food products. To ensure that products with a lower sodium content remain appealing to consumers, sodium reduction in food products must be conducted in a manner that does not lead to loss in product quality (e.g., preservation and taste properties). A straightforward approach is gradual reduction of the sodium content. However, if done too quickly, the reduction of sodium in food products without adjustments for the loss in saltiness can result in consumers switching to other products higher in sodium or compensating the taste difference by adding back sodium during preparation or consumption [12]. Therefore, other techniques that can reduce sodium while compensating either fully or partially for the saltiness taste are being explored. Sodium replacers can help to reduce sodium intake on a shorter term, while gradually weaning consumers off higher sodium levels, also known as habituation. Both techniques have advantages, disadvantages, and timelines for implementation [13,14]. Currently, potassium chloride is one of the most commonly used sodium chloride replacers as it has a good ability to convey the perception of a saltiness taste in food products [15,16].

Potassium chloride is a naturally occurring mineral salt, which is obtained from rock and sea salts in a manner similar to the extraction of sodium chloride. Dietary intake of potassium is associated with a decreased risk of hypertension, the opposite effect to sodium [17,18]. While the intake of sodium is too high, the average global intake of potassium is below the WHO guidelines (at least 3510 mg of potassium daily [19]). This makes potassium chloride an interesting salt replacer from a consumer, production, and, potentially, also from a health point of view. However, potassium chloride cannot be used in unlimited quantities as at higher levels it loses its ability to convey saltiness and can have an off-taste, often described as bitter, chemical and metallic. Depending on the format of the food, different levels of potassium chloride have been used to replace sodium chloride without compromising on sensorial aspects. For example, in watery solutions the off-taste of potassium chloride can initially be perceived at a concentration of 20% [20]. In pizza crust, replacement of 25% has been documented as possible [21], in brown and white bread 30% [22,23], in cheddar cheese 46% [24] and in feta cheese even up to 50% [25].

The safety of oral consumption of potassium chloride is supported by the natural occurrence of potassium in foods. As a result, potassium chloride has gained regulatory acceptance for use in food products in the United States and European Union and numerous other international scientific bodies and regulatory authorities [26,27,28,29,30]. Expert bodies agree that increasing potassium consumption from food in the population poses little risk for adverse effects [26,27,30]. While there is currently no established upper limit for potassium intakes, based on estimates of current intakes in European countries, the European Food Safety Authority (EFSA) states that the risk of adverse effects from potassium intake from food sources at 5000–6000 mg/day is considered low for the generally healthy population. Moreover, long-term intake of potassium supplements at levels of 3000 mg/day on top of usual intake from foods is also considered low risk for the general healthy adult population [31]. A recent study estimated the potassium intake for in the United States, Mexico, France and United Kingdom is 80%, 95%, 77% and 95%, respectively, below the WHO guideline for potassium [32].

The use of potassium chloride as a technique to reduce sodium in food products is expected to increase in the coming years. Therefore, it is important to understand the potential impact that increased industry-wide use of potassium chloride could have on the diet of the general population. To date this has not being examined, using population-based food intake data, we modeled the potential dietary impact of reformulating food products to lower sodium levels using potassium chloride. Three reformulation scenarios were examined, ranging from realistic to extreme, to allow the evaluation of: (1) How much potassium would be added to the diet through reformulation, and the safety of this level; (2) the impact on the general population’s compliance to the WHO guideline for potassium; and (3) in which food groups reformulation has the largest impact on potassium intake.

## 2. Materials and Methods

### 2.1. Population and Dietary Data

Food intake data were obtained from the Dutch National Food Consumption Survey (DNFCS) 2007–2010 [33]. This survey was conducted by the Dutch National Institute for Public Health and the Environment (RIVM) in a representative sample of the Dutch population. The dietary intake data were based on two non-consecutive 24-h recalls, which were completed by 3819 subjects. Results were weighted to adjust for variances in sociodemographic characteristics, seasonal and weekday differences. Sodium and potassium levels of all foods consumed in the survey were obtained from the Dutch Food Composition table (NEVO) table, 2011 [34]. Analyses were conducted using data from subjects aged between 18 and 65 years (*n* = 2106). Table 1 describes the characteristics of the study population and relevant baseline nutrient intakes. As nutrient intake distributions are skewed to the right, median intakes and the interquartile range (IQR) are used throughout this study for these variables. Subjects with high sodium, potassium or energy intakes were left in analyses to ensure that potential health and safety implications were addressed appropriately for all scenarios.

### 2.2. Food Product Group Classification

All of the 1772 foods consumed in the survey were classified into product groups in line with a set of proposed product-group based sodium criteria, which sets desired sodium levels per specific product groups with the aims towards achieving a maximum daily sodium intake of 2000 mg per day (5 g salt) [4]. For example, “meal sauces” are set a maximum sodium criteria of 340 mg/100 g and “cheese products” are allowed a maximum sodium content of 675 mg/100 g. These sodium criteria were then used in the modeling to indicate how much sodium should be removed from the product to comply with the guidelines. As not all foods can be reformulated, for example the sodium present in fresh fruit and vegetables cannot be removed, these types of products were not adjusted in the reformulation scenarios. Seventy-seven percent of the food products that were consumed in the survey were classified as foods in which sodium could potentially be reduced by reformulation. For example, none of the items in the food group “fresh fruit and vegetables”, “fresh fish” and “water” were adjusted, whereas all items in food groups such as “processed meat”, “processed fish”, “cheese products”, “soups” and “snacks” were adjusted if their sodium levels were higher than the maximum sodium criteria set for these food product groups. In some food product groups, individual exceptions were made; for example, in “milk products”, unflavored liquid milk and buttermilk were not adjusted, because in these products the sodium is not added during processing. A full list giving an overview of all foods consumed in the survey and indicating which food products were adjusted in the modeling is provided in the supplementary material.

### 2.3. Reformulation Scenarios

Three reformulation scenarios were developed to evaluate the impact of using potassium chloride as a sodium chloride replacer in food products. In the scenarios each food product was evaluated to see how much sodium chloride needed to be removed to meet the aforementioned product-specific sodium criteria [4]. For example, how much sodium should be removed in a meal sauce to reach the product criterion of maximum 340 mg/100 g was calculated. We then performed three reformulation scenarios. In the first scenario, up to 20% of the removed sodium was replaced by potassium chloride, as this level represents the minimum amount of sodium chloride that should be replaced with potassium chloride without any negative effects on taste due to potassium chloride. In the second scenario, up to 50% was replaced, which represents a scenario that may be used in certain products groups with higher sodium levels, and where a higher salty taste is expected by consumers. Finally, a 100% scenario, where all removed sodium was replaced, was performed to establish the most extreme replacement scenario. Although this scenario will never be applied by industry in practice, as it would lead to taste and preservation issues, it is important to show the most extreme impact. The added potassium chloride was calculated by multiplying the difference between the removed sodium chloride and the original sodium multiplied by 1.3, which is the correction factor for the difference in molecular weight between sodium chloride (58.44 g/mol) and potassium chloride (74.55 g/mol). Foods that were excluded from reformulation (as defined earlier) and foods in which the original sodium level was already below the criterion were left unchanged.

### 2.4. Data Analyses

For each scenario the amount of added potassium to the diet after simulating reformulation was calculated per individual. Box plots were used to provide insight in the population distribution. The box represents the median and IQR, the dots outside 1.5 times the IQR are suspected outliers. Furthermore, the 99th percentile of the population was calculated for safety estimations. Additionally, the potential impact of the reformulation scenarios was examined on compliance with the WHO potassium intake guideline for the total population. In doing so, the median intake and proportion of the population currently compliant to WHO guideline was calculated at baseline and after the application of the three reformulation scenarios. Finally, we calculated which food groups contributed the most to dietary potassium intake, both before and after application of the three reformulation scenarios. Modeling scenarios were performed with the DaDiet dietary modeling software from Dazult software [35]. Statistical analysis, like medians, boxplots and compliant proportions were performed with the statistical software package JMP [36].

## 3. Results

### 3.1. Reformulation Scenarios

**Figure 1:**
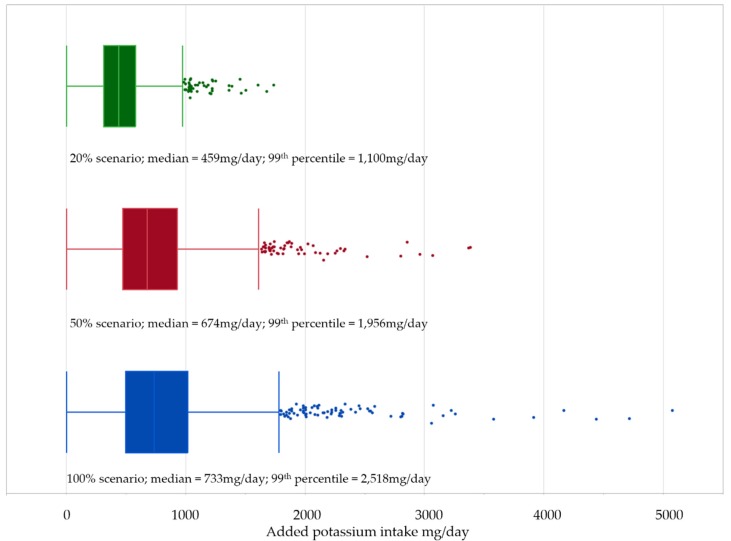
Intake distribution, combined for men and women showing the level of potassium added for each of the three reformulation scenarios.

Figure 1 shows the amount of potassium that was added to the diet due to reformulation in the three reformulation scenarios. In the 20% scenario the median added potassium was 435 mg/day (IQR = 268 mg/day), in the 50% scenario the median added potassium was 674 mg/day (IQR = 457 mg/day) and in the 100% scenario the median added potassium was 733 mg/day (IQR = 517 mg/day). All scenarios resulted in a small number of outliers; the 20% scenario showed 44 outliers (2.1% of the population), while the 50% and 100% scenario had 58 (2.8%) and 77 outliers (3.7%), respectively.

### 3.2. Impact of Reformulation on Intake

**Figure 2:**
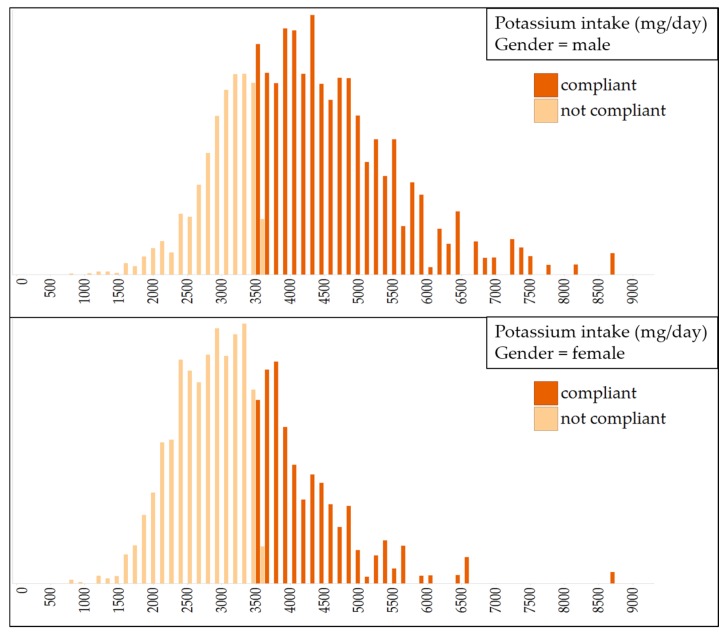
Baseline potassium intake in Dutch adult men and women (light bars represents proportion of population not compliant to the WHO guideline (3510 mg/day for both genders); dark bars represents the compliant proportion.

Figure 2 shows the baseline intake of potassium before the reformulation scenarios were applied. At baseline the median population potassium intake was 3334 mg/day (IQR = 1434 mg/day). The compliance to the WHO potassium intake guideline (3510 mg/day) was 44%. A gender difference was observed, whereby 38% of the male population and 70% of the female population had intakes below the guideline. The gender difference was explained by male and female differences in energy intake: the median daily potassium intake per calorie was 1.19 and 1.20 mg potassium/Kcal for males and females respectively.

**Figure 3:**
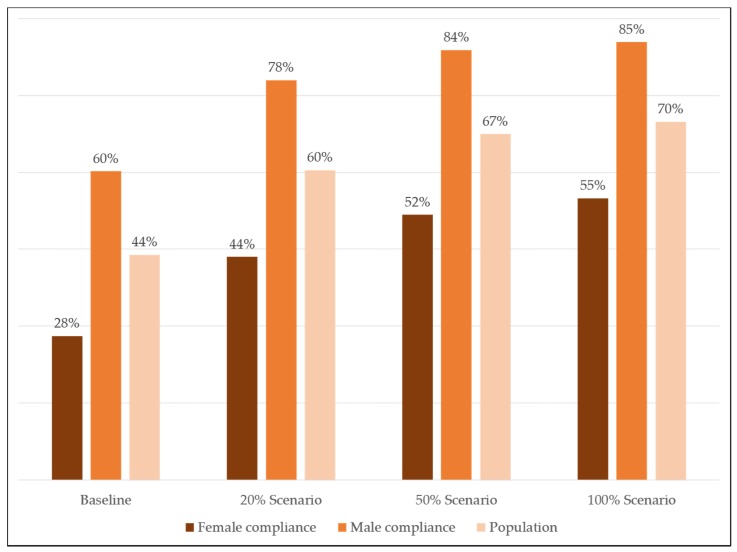
Proportions of Dutch adult men and women compliant with WHO potassium guideline at baseline and after each reformulation scenario.

Figure 3 shows the change in compliance to the WHO guideline for potassium from the baseline intake and during the application of the three reformulation scenarios. At baseline population compliance to the WHO guideline was 44%. After applying the reformulation scenarios compliance increased to 60%, 67% and 70%, for the 20%, 50% and 100% reformulation scenarios respectively. After simulated reformulation, the median potassium intake in the 20% scenario was 3805 mg/day, in the 50% scenario median intake was 4059 mg/day (IQR = 1694 mg/day) and in the 100% scenario median intake was 4126 mg/day (IQR = 1748 mg/day). Again, a gender difference in potassium compliance was observed, even after adjusting for energy intake. For men the median daily potassium intakes per calorie were 1.67, 1.79 and 1.81 mg potassium/Kcal for the three scenarios, respectively. For women, the median daily potassium intakes per calorie were 1.76, 1.83 and 1.90 mg potassium/Kcal.

### 3.3. Impact of Reformulation per Food Group

In the baseline diet, the top three food groups that contributed most to daily potassium intake were: “beverages”, “milk products” and “potato/pasta/rice”. Table 2 list shows the relative contribution of the food groups to the total potassium intake in the Dutch diet.

**Figure 4:**
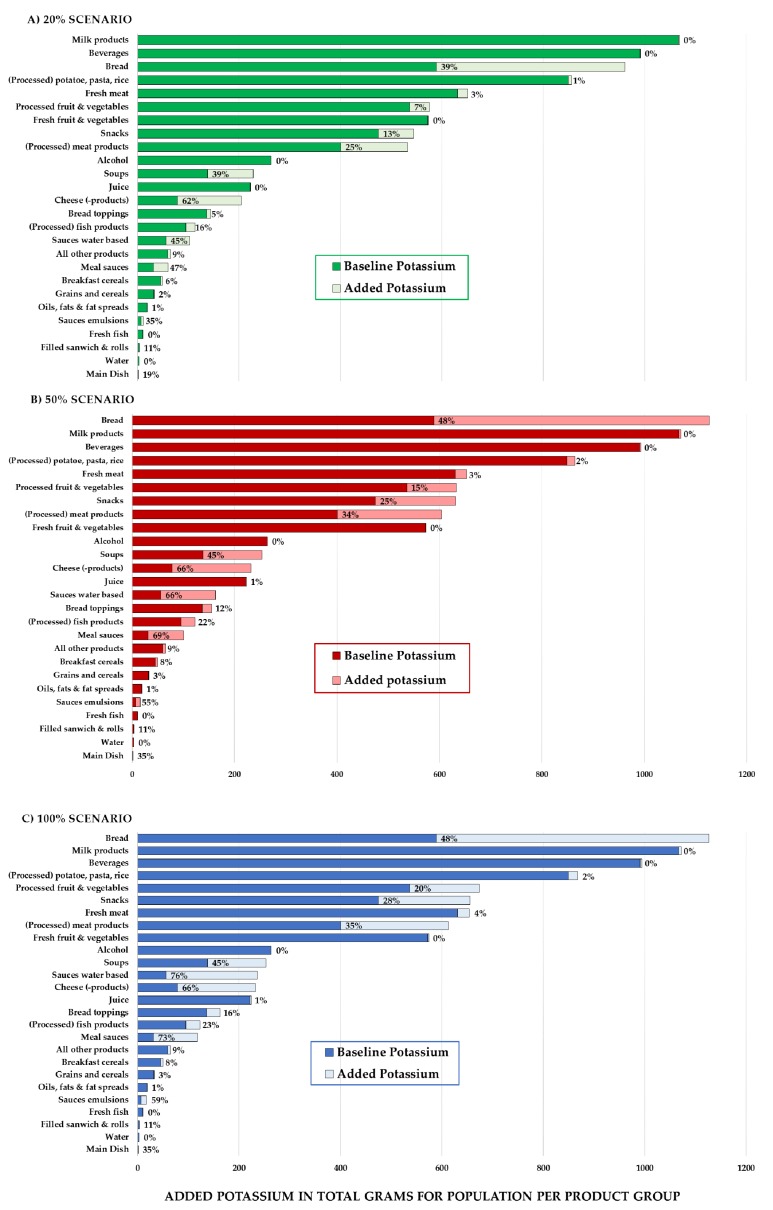
Level of potassium per food group shown at baseline (dark bar) plus the amount added during reformulation (light bar) for all three replacement scenarios (*n* = 2106).

To understand in which food groups the three reformulation scenarios showed the largest impact, we calculated the added potassium for each individual food group per scenario. Figure 4 shows per product group the baseline amount of potassium (dark bars) and the amount of added potassium after applying the reformulation scenarios (light bars). In the 20% scenario, it was clear that the replacement of sodium by potassium had the biggest impact on the “bread” product category. The average potassium intake from bread increased by 39%, which resulted in “bread” becoming the third largest contributor of potassium intake to the Dutch diet. Intakes from “milk products” and “beverages” were not noticeably changed by this scenario, but these groups remained the main contributors of potassium in the diet. Considerable changes in other main contributing groups such as “processed meat products”, “snacks”, and “processed fruit and vegetables” were also observed. Interestingly, a substantial increase in intake contribution in this scenario was also seen for “soups”, “cheese products”, “water based sauces” and “meal sauces”, which all initially played a small role in the dietary intake of potassium. In the 50% scenario, the average intake of potassium from “bread” increased to 48%, and notably “bread” became the main contributor of potassium in the diet. The role of bread did not change in the 100% scenario, which implies that in bread there are little or no products where more than 50% of the sodium was replaced. The largest differences between the 50% and 100% scenario were in “processed fruit and vegetables”, “snacks”, “cheese products”, “water based sauces” and “meal sauces”, which implies that in these in these groups the most frequently consumed products contain higher levels of sodium as compared to the other groups.

## 4. Discussion

The aim of the current study was to investigate the potential dietary impact of using potassium chloride as a sodium chloride replacer in the general population. This modeling study showed that replacing sodium chloride with potassium chloride in food products via three reformulation scenarios resulted in acceptable increases in the intake of potassium and moreover, that this can even help to increase population compliance to the WHO guideline for potassium.

Replacing sodium chloride via the three reformulation scenarios resulted in a median increase in potassium intake of 453 mg/day in the lowest reformulation scenario up to 733 mg/day in the most extreme replacement scenario. In the 20% scenario this is slightly less than the amount of potassium available in one medium sized banana and in the maximum scenario this is the equivalent of eating 1.5 bananas (374 mg potassium/100 g (NEVO) [34]; average portion size of 160 grams). This is within the limits that are considered by EFSA to be safe for the general healthy population, even in the most extreme 100% replacement scenario [31]. Therefore, these results imply that this technology can be safely used, even if it is applied industry-wide in the general healthy population. However, for some small patient populations with impaired potassium excretion it is argued that increasing potassium levels in foods could increase risk of hyperkalemia. These include diabetes, chronic kidney disease, end stage renal disease, severe heart failure, and adrenal insufficiency. As for these specific patient populations, there is no consensus regarding an upper tolerable limit for potassium, clear labeling of the use of potassium is important, although, as indicated, the amount of added potassium per product as well as at a total dietary intake level stays within normal dietary ranges.

Globally, in the general healthy adult population, potassium intakes are largely below the WHO guideline of minimum 3510 mg/day [19]. In this current population, the baseline level of compliance to WHO potassium guideline was 44%. Applying the 20% scenario would increase intake compliance by 16%. This scenario represents the level of replacement where negative sensorial issues are expected to be minimal in all product groups. Higher levels of reformulation have been applied in certain product groups, the highest reported level, 50% was reported in cheese products [24,25]. However, it is unlikely that this scenario can be applied to all food groups without negative sensorial issues. Applying the 50% scenario for all product groups would increase compliance by 23%. After reformulation a larger increase in compliance was observed in men. No large gender differences were observed in the potassium contribution from the different food groups, with the exception of fresh fruit and vegetables The observed gender differences after reformulation may be due to women having a higher contribution from potassium from fruit and vegetables, where little or no potassium was added, and men consuming more sodium rich foods that are able to be reformulated. As current evidence indicates that a reduction in dietary sodium and an increase in dietary potassium results in decreased incidence of hypertension [3], reformulating food products to lower sodium levels using potassium chloride may actually result in positive health effects in the general population. While this should not be used as the only initiative to increase potassium intake in the general population, it is a positive side benefit from this technology.

There is increasing evidence on the importance decreasing sodium intakes in combination with increased potassium intakes, and the sodium/potassium ratio [18]. Replacing industrially added sodium chloride by potassium chloride as shown in our modelling scenarios could have a positive contribution to obtaining a “healthy” ratio at the population level. Early studies suggest a ratio of approximately one to one would be beneficial for health [19]. However, WHO and other regulatory bodies have not yet established an optimal ratio. When an optimal ratio is established, future impact modelling should explore the potential dietary impact of using potassium as a sodium replacer. Furthermore, future research may also attempt to quantify the specific health benefits that reformulation with potassium can convey, however to date there is not enough quantitative evidence from intervention studies that allows the direct quantification of these benefits [37,38].

We observed a large variation in potassium contribution of different food groups at baseline. Replacement of sodium chloride by potassium chloride in bread had the biggest dietary impact on potassium intake. In previous studies on bread reformulation, a 30% replacement of sodium chloride by potassium chloride has been reported to be sensorially acceptable, therefore indicating that the two realistic scenarios could be feasibly applied [22,23]. Other major contributing food groups with a considerable impact at this level of change are processed fruit and vegetables, snacks and processed meat. In certain product groups where the level of replacement may go up as high as 50%, potassium intakes from the aforementioned product groups will increase even further. At this level we observed that soups, cheese, water based and meal sauces will start to have a substantial increase in impact on potassium intake as well. It is questionable whether in practice the sodium can be replaced for all products in these groups at this high level without sensorial or other technical limitations.

The current study is the first to date which has modelled the potential impact of potassium chloride as a sodium chloride replacer using complete dietary food consumption survey data. Some limitations need be taken into account when interpreting the results. First, the dietary intake data used in the current study is from 2010. Although this is the most recent data available in the Netherlands, some industry segments may have lowered their salt content since 2010, which may have already improved the current situation for both sodium and potassium. Second, the intake data in the survey does not include sodium or potassium intake from discretionary salt. However, for sodium this will not affect our conclusions as we are only looking at sodium intake from manufactured foods where sodium can reasonably be replaced by potassium [39] and this will not largely affect the outcomes regarding potassium intake as the intakes from potassium at a discretionary level is expected to be low. Furthermore, our study highlights the situation in a Western country, where the majority of sodium intake is coming from consumption of manufactured foods, and potassium intakes are below recommendations. Moreover, it is also noted that in the Netherlands, the potassium intake is relatively high compared to other Western countries [40], even though the majority of the Dutch population remains below potassium recommendations. In other countries where potassium intake is lower, reformulation may have a lower impact on increasing compliance towards the guidelines, but also therefore a lower chance of breaching safety concerns due to excessive intake. This is also the case for non-western countries where sodium intake is largely derived from discretionary salt, for example China [39].Here, the approach of replacing sodium by potassium in manufactured foods will have only show a small increase in potassium compliance. Nevertheless, it is likely that replacement of sodium chloride with potassium chloride will increase potassium guideline compliance without exceeding the EFSA safety recommendations also in these countries.

The addition of potassium chloride is currently the most commonly applied technique to replace sodium chloride in foods while maintaining a similar saltiness perception. The current study shows that when using realistic reformulation scenarios using potassium as a sodium replacer poses little risk of adverse effects in the general healthy adult population. It is a useful sodium reduction tool that if applied industry-wide, would not lead to excessive intakes of potassium in the general population. However, wide application of potassium chloride also needs to take into account sensorial properties of the mineral. As mentioned earlier, if used at too high levels potassium chloride can give a metallic off taste and it can lose its ability to contribute to the saltiness perception. Therefore, good product development through further research and development is essential in ensuring high product quality in reformulated foods.

In conclusion, this is the first modeling study that has estimated the dietary impact of added potassium chloride when used as a tool to reduce the sodium content of foods. Replacement of sodium chloride by potassium chloride, particularly in key contributing products according to current product targets, would result in better compliance to the WHO potassium intake guideline and not exceed EFSA safety recommendations. Potassium chloride has gained regulatory acceptance for use in food products in the United States and European Union and also numerous other international scientific bodies and regulatory authorities [26,27,28,29,30]. Therefore, based on the intake data, reformulation scenarios and increasing global regulatory acceptance, potassium chloride is recommended as a valuable, safe replacer for sodium chloride in foods products.

## Figures and Tables

**Table 1:** Baseline characteristics of subjects.

Characteristics	Men (*N* = 1048)	Women (*N* = 1058)	Total (*N* = 2106)
**Age (years)**			
Mean	39	39	39
Standard deviation	15	15	15
Minimum	18	18	18
Maximum	65	65	65
**BMI (kg/m^2^)**			
Mean	26	25	25
Standard deviation	4	6	5
Minimum	16	14	14
Maximum	49	57	57
**Energy intake at baseline (kcal/day)**			
Median	2619	1911	2230
IQR	964	712	990
Minimum	418	279	279
Maximum	8139	4658	8139
Sodium intake at baseline (mg/day) *			
Median	3101	2302	2649
IQR	1304	1060	1310
Minimum	400	331	331
Maximum	8726	6068	8726
**Potassium intake at baseline (mg/day)**			
Median	3789	2946	3334
IQR	1485	1204	1434
Minimum	804	704	704
Maximum	8798	8723	8798

Note: * this excludes discretionary salt intake.

**Table 2:** Food groups contributing to potassium intake at baseline, split by gender.

Food Group Men		Women	All
Milk products	14.5%	14.7%	14.6%
Beverages	13.4%	13.7%	13.6%
(Processed) potato, pasta, rice	12.6%	10.4%	11.6%
Fresh meat	8.9%	8.3%	8.6%
Bread	8.2%	7.9%	8.1%
Fresh fruit and vegetables	6.2%	9.9%	7.8%
Processed fruit & vegetables	6.8%	8.0%	7.3%
Snacks	6.2%	6.9%	6.5%
(Processed) meat products	6.1%	4.7%	5.5%
Alcohol	4.8%	2.1%	3.6%
Juice	2.7%	3.5%	3.0%
Soups	1.9%	1.9%	1.9%
Bread toppings	1.9%	1.8%	1.9%
(Processed) fish products	1.2%	1.4%	1.3%
12 remaining food groups	4.6%	4.9%	4.7%

## Supplementary Materials

The following are available online at http://www.mdpi.com/2072-6643/8/4/235/s1. Table S1: Foods and food groups.

## Abbreviations

The following abbreviations are used in this manuscript:

DNFCS	Dutch National Food Consumption Survey
EFSA	European Food Safety Authority
IQR	Interquartile range
NEVO	Nederlands Voedingsstoffenbestand (Dutch Food Composition Database)
RIVM	Rijksinstituut Voor Volksgezondheid en Milieu (Dutch National Institute for Public Health and the Environment)
WHO	World Health Organization
